# Determination of *in situ* degradation parameters and feeding level of pineapple (*Ananas comosus* L.) cannery by-product to Hanwoo steers

**DOI:** 10.5713/ajas.20.0083

**Published:** 2020-08-24

**Authors:** Yongjun Choi, Keunkyu Park, Sangrak Lee, Youngjun Na

**Affiliations:** 1 Department of Animal Science and Technology, Konkuk University, Seoul 05029, Korea

**Keywords:** Beef Cattle, By-product, Feeding Level, Pineapple, Hanwoo, Steer

## Abstract

**Objective:**

The objectives of this study were to determine the *in situ* degradation parameters and appropriate feeding level of pineapple cannery by-products (PCB) based on the growth performance and blood parameters of growing Hanwoo (*Bos taurus coreanae*) steers fed various levels of PCB.

**Methods:**

Two ruminally cannulated Holstein cows were used for *in situ* disappearance rate measurements. Nylon bags (5×10 cm, 45 μm pore size) filled with 5 g of PCB in triplicate were inserted into the ventral sac of the two cannulated Holsteins cows and incubated for 0, 2, 4, 8, 16, 24, and 48 h. A total of 16 castrated growing Hanwoo steers (12.5±0.5 months old, 302.9±25.7 kg of initial body weight [BW]) were used for the experiment. Animals were stratified by initial BW and then randomly assigned to one of four experimental diets (0%, 1.5%, 3.0%, or 6.0% of PCB, on the dry matter [DM] basis) fed for 91-d, including 30-d of adaptation.

**Results:**

Soluble fraction *a* of DM and crude protein (CP) was 61.9% and 86.0%, fraction *b* of DM and CP was 32.7% and 11.2%, and indigestible fraction *c* of DM and CP was 5.4% and 2.8%. The 6.0% PCB feeding group showed lower productivity compared to animals in the other treatments. Increasing the dietary level of PCB did not alter DM intake, but it was numerically lowest in the 6.0% feeding group. The gain to feed ratio was linearly decreased by increasing of PCB. The quadratic broken-line test estimated that 2.5% (DM basis) was the maximum feeding level of PCB in growing Hanwoo steers (y = 0.103 − 0.001×[1.245−x]^2^, R^2^ = 0.18).

**Conclusion:**

Diets containing up to 2.5% PCB can be fed to growing Hanwoo steers without adverse effects on growth performance.

## INTRODUCTION

The use of human-inedible by-products as feed ingredients can potentially diminish the dependence on grains and pulse crops in the livestock industry [1] as well as solve environmental problems associated with improper waste disposal [2]. Pineapple (*Ananas comosus* L.) is widely grown in tropical and subtropical regions and it has been processing large quantities of fresh pineapple fruit as slices, chunks, crush, jam, or juice. Among these, canned fruit is the main product of the pineapple industry worldwide [3]. Because only part of pineapple fruit is generally utilized for cannery processing, pineapple processing factories generate considerable amounts of by-products, such as skin, crown ends, bud ends, and cores [4]. Although pineapple cannery by-products (PCB) do not completely meet the nutrient requirements of animals as a sole feed source [5], they have typically been used as a feed ingredient in the diets of ruminants, swine, poultry, and fish [6]. Although previous studies have sought to determine the effect of PCB on the growth performance of beef cattle [7–9], most of these studies were conducted using survey methodology rather than by performing specifically designed animal experiments. Consequently, there remains a need for further research to determine the appropriate feeding level of PCB in beef cattle diets. Therefore, the objectives of the present study were to determine i) the effect of PCB level on the growth performance and blood parameters of growing Hanwoo (*Bos taurus coreanae*) steers, and ii) the appropriate level of PCB in beef cattle diets.

## MATERIALS AND METHODS

### Animal care

The experiment was carried out in an experimental facility of Konkuk University at Chungju-si, Chungcheongbuk-do, Korea (latitude 36.99° and longitude 127.93°). All research protocols were approved by Konkuk University Animal Care and Use Committee (Approval number: KU16139).

### Pineapple cannery by-product

**Figure 1:**
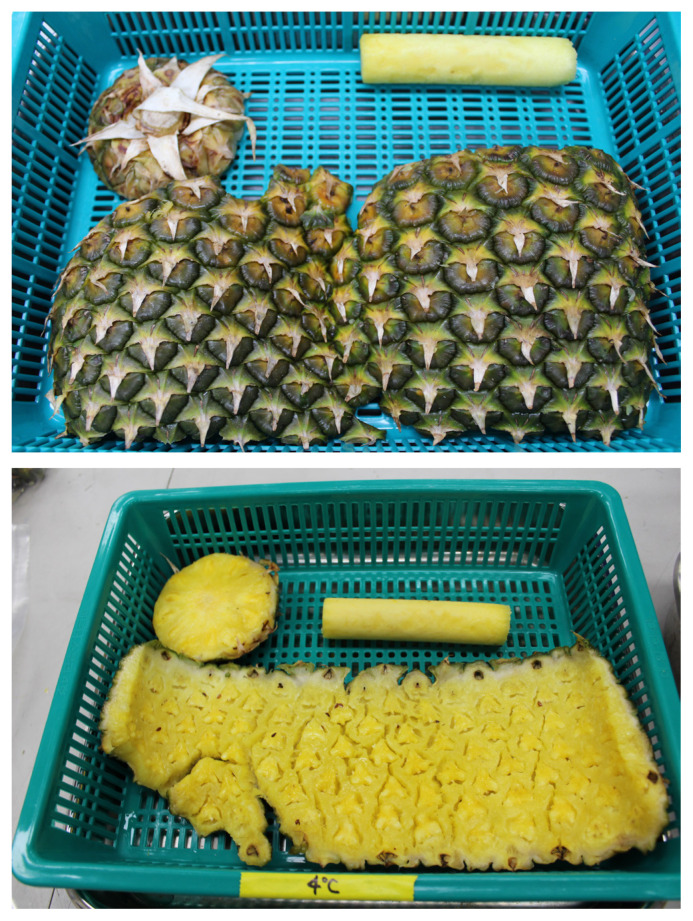
Photos of the skin, bud end, and core of pineapple cannery by-product generated from food processing factory.

The PCB was obtained from Shinsegae Food Processing Facility (Icheon, Korea). The PCB which used in the current analyses contains the skin, crown ends, bud ends, and cores (Figure 1). The composition of lipids and amino acids was presented as Supplementary Table A1 and A2. The PCB was discharged about 165 tons per month in the facility, it has been stored in a container with a leachate outlet in room temperature. The PCB used in the experiment showed a pH of 3.90 with the leachate removed (Figure 1). The test was performed that changes in properties of PCB by time (every day for 7 d) and temperature (0°C, 23°C, and 30°C) before formulated to experimental feed. In the temperature of 23°C and 30°C, the flavor and surface color of the PCB was changed after 5 d, the fungus appeared after 7 d, and it did not show appearance change at 0 d except moisture evaporation. The PCB was used for experimental feed within 5 days after dumped based on the test result.

### *In situ* procedure

Two ruminally cannulated Holstein cows were used for *in situ* disappearance rate measurements. The cows were fed commercial concentrate pellets (dry matter [DM], 92.3%; crude protein [CP], 14.5% of DM; neutral detergent fiber [NDF], 31.8% DM; acid detergent fiber [ADF], 11.6% DM; ether extract [EE], 3.7% DM; ash, 7.5% DM) and rice straw (DM, 92.1%; CP, 5.5% DM; NDF, 62.1% DM; ADF, 36.6% DM; EE, 1.4% DM; ash, 5.2% DM) with *ad libitum* access to feed during the experiment. The PCB samples were dried and milled to pass through a 3-mm screen (Wiley Mill; Thomas Scientific, Swedesboro, NJ, USA). The DM, ash, CP, EE, ADF, NDF, non-fiber carbohydrate (NFC), and water-soluble carbohydrate (WSC) concentrations in PCB were 9.2%, 4.9%, 5.7%, 1.2%, 17.7%, 39.4%, 48.8%, and 5.4% of DM, respectively. Nylon bags (5×10 cm, 45 μm pore size; R510, ANKOM Inc., NY, USA) filled with 5 g of PCB in triplicate were inserted into the ventral sac of two cannulated Holsteins cows and incubated for 0, 2, 4, 8, 16, 24, and 48 h according to NRC [10]. After incubation, the nylon bags were rinsed in tap water, dried at 60°C, and then weighed for DM and CP analysis. Degradation was assessed using the formula of Ørskov [11]:

$$\text{P}=a+b\;(1-e^{-ct}),$$

where P is the actual degradation after time t; *a* is the intercept of the degradation curve at time zero; *b* is the potential degradability of the component of the protein which will, in time, be degraded; *c* is the rate constant for the degradation of *b*; and *t* is time. For estimation of the fraction *b*, regression analysis was performed using SAS PROC REG (Version 9.4, SAS Institute Inc., Cary, NC, USA).

The effective degradability (ED) of DM and CP was calculated using the following equation:

ED=a+(b×c)/(c+k),

where *k* is the estimated rate of outflow from the rumen and *a*, *b*, and *c* are the same parameters as described above. The ED was estimated as ED2, ED5, and ED8 assuming rumen solid outflow rates of 0.02, 0.05, and 0.08/h, which are representative of low, medium, and high feeding intake, respectively.

### Animals and experimental design

Sixteen growing Hanwoo (*Bos taurus coreanae*) steers (12.5± 0.5 months old, 302.9±25.7 kg of initial body weight [BW]) were used for the experiment. All steers were vaccinated for foot and mouth disease before the experiment. Animals were stratified by initial BW and then randomly assigned to one of four experimental diets. Each pen was equipped with individual electronic feeding gates for individual feeding. The experimental diets contained 0%, 1.5%, 3.0%, or 6.0% DM of PCB (Table 1) and it was formulated every week for the experiment. The experimental unit was an individual animal. Experimental diets were formulated to meet or exceed the nutrient requirements of the Korean Feeding Standard for Hanwoo and daily weight gain of steers was predicted 0.8 kg/d on the control diet (Presented on the basis of 300 kg of BW, 7.1 kg of DM intake (DMI), and total digestible nutrients value 68.0) [12]. Animals were fed twice a day at 09:00 h and 17:00 h *ad libitum*, with 5% to 10% refusals. The experiment was continued for 91 d, including 30 d for diet adaptation and 61 d for data collection. The BW (unshrunk basis) was measured before morning feeding at d 0, 30, 60, and 91 of the experimental periods. Offered diets and orts were weighed daily for DMI measurement.

### Chemical analysis

All samples were dried and ground to pass through a 1-mm screen (Wiley Mill; Thomas Scientific, USA). All samples were dried in an air drying oven at 60°C for 48 h to analyze DM [13]. Ash (method 942.05), CP (method 990.03), and EE (method 920.39) were analyzed in duplicate according to the standard methods of the AOAC [13]. ANKOM fiber analyzer 200 (ANKOM Technology Crop., Macedon, NY, USA) was used for NDF (method 2002.04) and ADF (method 973.18) analyses. The NFC content of feeds was calculated by subtraction of CP, NDF, EE, and ash from 100.

### Blood sampling and analysis

Blood samples were collected at d 70 and d 90 of the experiment for serum analysis. Blood samples (15 mL) were collected via the jugular vein using 18-gauge needles and transferred to silicon-coated serum tubes (Vacutainer, BD, Franklin Lakes, NJ, USA). Serum samples were separated by centrifugation at 2,000×*g* at 4°C for 20 min and stored at −80°C until subsequent analysis. These samples were analyzed using an automated chemical analyzer (Model 7180 Clinical Analyzer, Hitachi Ltd, Tokyo, Japan). Reagents for analyses of albumin, glutamic-oxaloacetic transaminase (GOT), glutamic pyruvate transaminase, blood urea nitrogen, creatine, triglycerides, high-density lipoproteins, low-density lipoproteins, calcium, inorganic phosphorus, magnesium, and non-esterified fatty acids were purchased from JW Medical (Seoul, Korea).

### Statistical analysis

Data were analyzed using SAS PROC MIXED (Version 9.4, SAS Institute Inc., Cary, NC, USA). The model included PCB level as the fixed effect. Orthogonal contrast for linear and quadratic effect was performed using polynomials determined by SAS PROC IML. Treatment effects were considered significant at p<0.05, and trends were considered at 0.05≤p<0.10. Quadratic broken-line regression analysis was conducted using SAS PROC NLIN [14]. Gain to feed (G:F) ratio was used as the response criterion.

## RESULTS

The DM and CP degradation parameters, and the ED values of PCB are presented in Table 2. Soluble fraction *a* of DM and CP was 61.9% and 86.0%, whereas fraction *b* of DM and CP was 32.7% and 11.2%, and indigestible fraction *c* of DM and CP was 5.4% and 2.8%.

**Figure 2:**
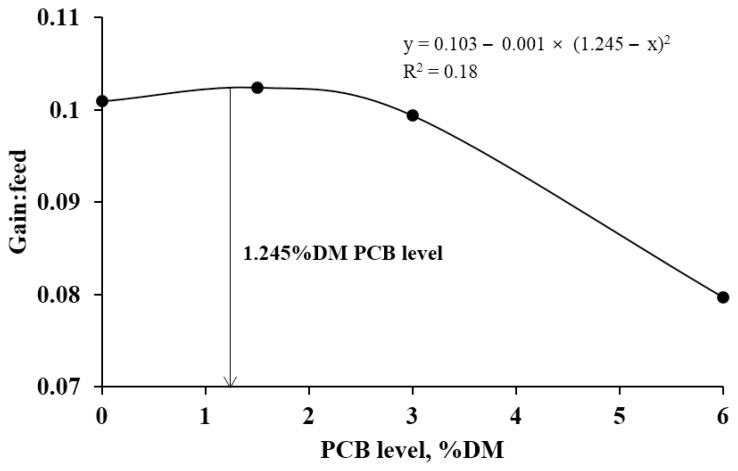
The quadratic broken-line analysis of gain:feed (G:F; R^2^ = 0.18; p = 0.283) as a level of pineapple cannery by-product (PCB) in growing Hanwoo steers (dry matter basis, n = 4 for each regression line).

There was a linear decrease in body weight gain (BWG; p = 0.006), average daily gain (ADG; p = 0.006), and G:F ratio (p = 0.008) with increasing of dietary PCB level (Table 3). The DMI tended to linearly (p = 0.051) decrease with increasing of dietary PCB level. As the level of the PCB increased, serum albumin concentration linearly (p = 0.010) decreased with increasing of dietary PCB level (Table 4). Serum GOT concentration was linearly (p = 0.025) increased with increasing of dietary PCB level. The quadratic broken-line test estimated that 3.25% DM (21.1%, on an as-fed basis) was the maximum feeding level of PCB for growing Hanwoo steers (y = 0.103+ 0.001×[1.245−x]^2^, R^2^ = 0.18; p = 0.283; Figure 2).

## DISCUSSION

Because the pineapple by-products generally contain high water-soluble and moisture contents like other fruit by-products, they have the potential to readily decay [15]. Although there are certain procedures for decreasing the moisture content of PCB, such as sun-drying or artificial drying, sun-drying is impracticable during the rainy season and artificial drying generates additional expenses and it also requires special facilities. It has long been known that the wet total mixed ration (TMR) feeding system is a relevant strategy when using high-moisture by-product ingredients such as brewer’s grain, citrus pulp, and grape pomace. However, little work has been conducted to determine the suitability of high-moisture PCB as an ingredient for TMR diets. Although PCB have been used as a roughage source replacer in some experiments because of their large amounts of fibrous contents [16,17], the high amounts of non-fibrous carbohydrate in PCB should also be considered because of its readily degradable characteristics in the rumen.

The degradation characteristics of feed ingredients are effectively used to anticipate rumen fermentation [11]. To our knowledge, only one scientific study has examined the *in situ* degradability of pineapple by-product [18]. However, it is difficult to directly compare the results of the present study with those of the previous study [18] because they used a mixture of PCB and elephant grass silage for the *in situ* procedure, whereas in the current study, we used PCB as the sole treatment. In addition, *in situ* procedures are affected by various factors such as pore size [19,20], test animal diet, and sampling schedules [21], or bag surface area ratio [22, 23]. Because the rapid rumen degradation results in rumen acidosis [24,25], PCB has to be used appropriately with consideration of their highly degradable DM and CP contents. Piao et al proposed that the synchronization of energy and nitrogen supply can improve the efficiency of rumen microbial protein synthesis [26]. Therefore, suggest that the rapidly degradable nitrogen sources to improve efficiency of the rumen fermentation should also be used in diets containing PCB due to their rapidly degradable characteristics. The kinetics of the ruminal degradation of PCB, which were estimated in the current study can provide values for more complex dynamic digestion models and subsequent animal trials.

Growth performance is directly affected by DMI, as a consequence of nutrient supply. In the current experiment, although DMI was not significantly affected by the dietary level of PCB, the numerically lowest value of DMI during the entire experimental period was observed for diets containing 39% PCB. Basically, voluntary feed intake and the productivity of ruminants might decrease due to the high moisture content if an excessive amount of PCB is used in the diet [27]. In the previous study, the DMI decreased linearly with an increasing level of pineapple by-product in the diet as a substitute for corn silage [9]. In general, PCB contains a high level of WSC and non-fiber carbohydrate contents. Previous studies have reported that although a high concentration of WSC in the diet can increase palatability [28,29], it can also decrease ruminal pH and the activity of fiber-using bacteria [30]. Therefore, it would appear that the rapid degradation characteristics of PCB in the rumen may alter the ruminal microbial community, thereby decreasing total nutrient digestibility. In addition, there was no significant difference in the growth performance of steers during period 1 (d 0 to 30), whereas in period 2, BWG and ADG decreased linearly with an increasing level of PCB. This indicate that the adverse effects of excessive feeding of PCB in steer diets could gradually increase with time [31].

There was reported that pineapple by-products can be used to replace 50% (as-fed basis) of roughage sources for dairy cattle ration without any adverse effect [32]. In a beef cattle trial, 30% to 100% (as-fed basis) of fresh pineapple by-products has been used as a roughage substitute in beef cattle diet in local production regions [4]. However, this study has certain limitations because the results are based on farm survey data, whereas specifically designed animal experiments are needed to determine the appropriate feeding level of PCB. To our knowledge, there has been only one specifically designed study that sought to determine the effect of pineapple by-product level in beef cattle except for previous experiment [31]. It determined that silage containing pineapple by-products have an effect on the growth performance of bulls [9]. These authors concluded that the DM, organic matter, and metabolized energy intake were linearly decreased when pineapple by-product silage was gradually increased (0% to 31% DM) in the diet as a substitute for corn silage whereas BWG and ADG were not affected. Although the results of this study indicated that pineapple by-product silage did not affect growth performance, it is difficult to directly compare these findings with those of the current study because Prado et al [9] used small breed animals (328 kg of initial BW at 20 months old), whereas we used animals of 302.9 kg of initial BW at 12.5 months old.

On the basis of the quadratic broken-line test performed in the present study, the recommended levels of the PCB for growing Hanwoo steer can be set at between 0% and 3.25% DM(FCR used as a criterion) in the diet. Although the findings of the present study provide a framework for the determination of appropriate levels of dietary PCB, further work is needed to establish the long-term effects, from growing phase to slaughter, of PCB on the growth performance and carcass traits of beef cattle.

In conclusion, the use of PCB in ruminant diets has to be carefully considered because they have high moisture and rapidly degradable DM and CP contents. In addition, rapidly degradable nitrogen sources should be included in diets containing PCB due to their high amounts of soluble carbohydrates. In conclusion, PCB can be fed at levels up to 3.0% DM of the steer diet without adverse effect on growth performance or blood parameters. However, considering the lower performance of the 6.0% DM PCB feeding group in the present study, an excessive level of PCB is not recommended. On the basis of the quadratic broken-line test, which uses the FCR as a response criterion, diets containing up to 3.25% DM PCB can be used for growing Hanwoo steers.

## Figures and Tables

**Table 1:** Ingredients and chemical composition of experimental diets for the growing Hanwoo steers

Items	Pineapple cannery by-product level (% DM)			

	0	1.5	3.0	6.0
Ingredients				
Pineapple cannery by-product (% DM)	0.0	1.5	3.0	6.0
Corn, cracked (% DM)	18.5	17.7	16.9	15.4
Soybean meal (% DM)	2.4	2.4	2.3	2.3
Corn gluten meal (% DM)	15.2	15.2	15.2	15.2
Wheat meal (% DM)	20.0	20.0	20.0	20.0
Rapeseed meal (% DM)	2.1	2.1	2.1	2.1
Wheat meal (% DM)	0.7	0.7	0.7	0.7
Coconut kernel meal (% DM)	0.7	0.7	0.6	0.6
Palm kernel meal (% DM)	1.3	1.3	1.3	1.3
Alfalfa, hay (% DM)	12.5	12.5	12.5	12.5
Klein grass, hay (% DM)	12.5	12.5	12.5	12.5
Annual rye grass, hay (% DM)	11.8	11.1	10.4	9.0
Limestone (% DM)	1.5	1.5	1.5	1.5
Salt (% DM)	0.3	0.3	0.3	0.3
Vitamin mineral premix1) (% DM)	0.4	0.4	0.4	0.4
Water (%)	35.1	26.4	17.6	0.0
Chemical composition				
DM (%)	59.8	59.8	59.8	59.8
Ash (% DM)	7.9	7.9	8.0	8.0
CP (% DM)	15.2	15.2	15.2	15.2
EE (% DM)	2.9	2.9	2.9	2.8
ADF (% DM)	21.0	21.0	20.9	20.7
NDF (% DM)	44.0	44.0	44.0	43.9
NFC (% DM)	30.0	30.0	30.0	30.0
TDN (% DM)	68.2	68.2	68.2	68.3

DM, dry matter; CP, crude protein; EE, ether extract; ADF, acid detergent fiber; NDF, neutral detergent fiber; NFC, non-fiber carbohydrate; TDN, total digestible nutrients.

1) Vitamin E, 500 IU/kg; Fe, 50,000 mg/kg; Cu, 6,720 mg/kg; Mn, 24,000 mg/kg; Zn, 30,000 mg/kg.

**Table 2:** *In situ* degradation parameters of pineapple cannery by-product

Items1)	Degradation parameter	

	Dry matter	Crude protein
*a* (% DM)	61.9	86.2
*b* (% DM)	32.7	11.2
*c* (h^−1^)	0.079	0.072
ED2 (% DM)	88.0	94.7
ED5 (% DM)	81.9	92.6
ED8 (% DM)	78.2	91.3

DM, dry matter.

1) *a*, the fraction of dry matter and crude protein at the initiation of incubation; *b*, the fraction of dry matter and crude protein insoluble but degradable in the rumen; *c*, the rate constant (h^−1^) of disappearance of fraction b; ED, effective degradability at three ruminal passage rates (i.e., 0.02, 0.05, and 0.08/h).

**Table 3:** Effect of pineapple cannery by-product on dry matter intake, body weight, average daily gain, and gain to feed ratio of the growing Hanwoo steers

Items	Pineapple by-product level (% DM)				SEM	p-value	
	
	0	1.5	3.0	6.0		Linear	Quadratic
Initial BW (kg)	297.6	311.9	303.9	298.4	14.0	0.861	0.563
Final BW (kg)	352.1	369.0	355.3	333.6	16.9	0.306	0.395
BWG (kg)	54.5	57.1	51.4	35.3	4.7	0.006	0.186
ADG (kg/d)	0.91	0.95	0.86	0.59	0.08	0.006	0.189
DMI (kg/d)	8.92	8.96	8.76	7.57	0.49	0.051	0.365
G:F ratio	0.10	0.11	0.10	0.08	0.01	0.008	0.170

DM, dry matter; SEM, standard error of the mean; BW, body weight; BWG, body weight gain; ADG, average daily gain; DMI, dry matter intake; G:F, gain to feed.

**Table 4:** Effect of pineapple cannery by-product on serum blood parameters of the growing Hanwoo steers (experiment 2)

Items	Pineapple cannery by-product level (% DM)				SEM	p-value	
	
	0	1.5	3.0	6.0		Linear	Quadratic
Albumin (g/dL)	3.7	3.6	3.6	3.3	0.10	0.010	0.431
GOT (U/L)	76.5	85.3	90.0	91.0	3.88	0.025	0.169
GPT (U/L)	30.5	29.8	33.5	30.5	1.71	0.799	0.341
BUN (mg/dL)	15.8	12.5	17.8	18.5	2.88	0.306	0.769
Creatine (mg/dL)	1.3	1.1	1.1	1.2	0.07	0.655	0.052
Triglycerides (mg/dL)	23.3	15.3	15.3	16.3	3.47	0.274	0.182
HDL (mg/dL)	205.8	201.0	193.5	198.5	11.17	0.641	0.571
LDL (mg/dL)	32.0	33.3	32.3	31.3	3.46	0.807	0.807
Calcium (mg/dL)	10.4	10.4	10.5	9.9	0.21	0.097	0.264
Inorganic P (mg/dL)	9.4	9.3	8.3	9.4	0.57	0.894	0.239
Mg (mg/dL)	3.0	3.0	2.8	3.0	0.10	0.791	0.147
NEFA (mmol/L)	145.7	155.7	147.2	206.1	33.83	0.223	0.562

DM, dry matter; SEM, standard error of the mean; GOT, glutamic-oxaloacetic transaminase; GPT, glutamic pyruvic transaminase; BUN, blood urea nitrogen; HDL, high-density lipoproteins; LDL, low-density lipoproteins; NEFA, non-esterified fatty acid.
